# Blood eosinophil counts and postoperative outcomes in early-stage lung cancer: a retrospective cohort study

**DOI:** 10.1186/s12931-026-03672-9

**Published:** 2026-05-01

**Authors:** Everglad Mugutso, Ophir Freund, Isabelle Pitrou, Ankita Ghatak, Cédric Roux, Samantha Jacobson, Jonathan-Raphaël Stetco, Sara Kutzkel, Amir Bar-Shai, Benjamin Smith, Nicole Ezer

**Affiliations:** 1 Research Institute McGill University Health Centre, Centre for Outcomes Research and Evaluation (CORE), 5252 De Maisonneuve, Montreal, QC H4A 3S9 Canada; 2 Tel-Aviv Sourasky Medical Center and Faculty of Medicine, The Institute of Pulmonary MedicineTel-Aviv University, Tel Aviv, Israel; 3 Bioinformatics Department, Research Institute McGill University Health Centre, Montreal, QC Canada; 4 University of Sherbrooke, Faculty of Medicine and Health SciencesSherbrooke, QC Canada; 5 Division of Respiratory Medicine, McGill University Health Centre, Montreal, QC Canada

**Keywords:** Blood eosinophil counts, Chronic obstructive pulmonary disease, Healthcare utilization, Inhaled corticosteroids, Lung cancer, Non-small cell lung cancer, Thoracic surgery, Outcomes

## Abstract

**Background:**

Lung cancer resection is curative but associated with postoperative morbidity and mortality. This study evaluated whether elevated blood eosinophil count (BEC) was associated with postoperative outcomes in early-stage lung cancer.

**Methods:**

This was a retrospective cohort study of consecutive adult patients undergoing lung resection for stage I and II non-small cell lung cancer in a large tertiary referral center from September 2017 to June 2021. Data were drawn from the institution’s Data Warehouse. The primary outcome was 90-day healthcare utilization defined as emergency department visit or hospital readmission. Secondary outcomes were postoperative complications, index hospitalization length of stay, and 1-year survival. Preoperative 90-day BEC was categorized by a threshold of 200 cells/µL. Covariates were age, sex, smoking status, Charlson Comorbidity Index, chronic obstructive pulmonary disease (COPD), asthma, tumor size, nodal status, surgical approach, and blood results (white blood cells, hemoglobin, and creatinine). The main analyses were validated by a second international cohort. Log-Poisson with robust variance estimation and Cox proportional hazards regression models were fit for primary and secondary outcomes. Analyses were replicated for BEC thresholds of 150 and 300 cells/µL.

**Results:**

Among 715 patients undergoing lung resection (median age = 69 years, 42% male, 29% with COPD), 146 patients (20%) had high preoperative BEC ≥ 200 cells/µL. BEC ≥ 200 cells/µL was associated with a higher rate of 90-day healthcare utilization:19% vs. 14% for BEC < 200 cells/µL. This association remained after adjustment (Risk Ratio [RR], 1.52; 95% Confidence Interval [CI], 1.02–2.25) and the validation cohort (RR, 2.23; 95% CI, 1.06–4.69). BEC as a continuous measure was also associated with the primary outcome in both cohorts: RR, 2.15 (95% CI, 1.49–3.12) and RR, 1.42 (95% CI, 1.10–1.94), respectively. BEC ≥ 200 cells/µL was associated with higher probability of death 1 year post-surgery (adjusted Hazard Ratio, 2.41; 95% CI, 1.08–5.35). There was no difference in the risk of postoperative pulmonary complications between high and low BEC (RR, 0.86; 95% CI, 0.58–1.27).

**Conclusions:**

Elevated preoperative BEC was associated with higher risk of postoperative healthcare utilization and lower 1-year survival after lung cancer resection among patients with or without respiratory disease.

**Supplementary Information:**

The online version contains supplementary material available at 10.1186/s12931-026-03672-9.

## Background

Advances in lung cancer screening have increased the number of patients eligible for lung resection with curative intent [1, 2]. Chronic obstructive pulmonary disease (COPD) exists as a comorbid condition in 50 to 70% of patients diagnosed with lung cancer [3–5], although frequently underdiagnosed and untreated [6, 7]. Patients with COPD and lung cancer have worse lung cancer-specific survival [8–10], and have a higher rate of respiratory complications, such as pneumonia and prolonged air leak [9, 11]. COPD is often punctuated by acute exacerbations [12], which also increase the risk of mortality [13, 14].

Eosinophilic inflammation is present in up to 30% of COPD exacerbations and 50% of asthma exacerbations [15, 16]. Bafadhel at al. identified four biologic clusters of COPD exacerbations with their respective biomarkers; blood eosinophil counts (BECs) being the biomarker of the eosinophil-associated COPD exacerbations type [17]. Patients with elevated BECs have a greater propensity for acute exacerbations of COPD and benefit most from inhaled corticosteroids (ICS) to reduce the risk of exacerbations [18, 19]. Acute exacerbations of COPD are associated with an increased all-cause and respiratory mortality at 1 year with an incremental response the higher the number of events [13, 14]. In the Canadian Cohort of Obstructive Lung Disease (CanCOLD) cohort study, high BECs were associated with a more rapid decline in forced expiratory volume in 1 s (FEV_1_) in the presence or absence of COPD [20]. BECs are used to guide therapy for both asthma and COPD as evidence has shown that patients with higher BECs have a better response to ICS [12, 18, 21, 22], and response to anti-interleukin (IL)-5 biologic therapy [15].

Despite the wealth of data on eosinophilia in COPD and asthma, studies investigating the significance of BECs in patients with early-stage lung cancer presenting for surgical resection are lacking [23]. A retrospective study demonstrated that blood eosinophilia was associated with poor outcomes including worse graft survival after lung transplantation [24]. Risk stratification is crucial in patients with early-stage lung cancer because of the high prevalence of COPD and its heterogeneity with potentially different impact on surgical outcomes. Whether BEC is a biomarker for patients with a propensity for a more complicated postoperative course is unknown. Additionally, whether higher BECs are associated with postoperative mortality is unknown. This study objective was to investigate if there is an association between BECs in patients with early-stage lung cancer undergoing resection surgery and postoperative surgical outcomes.

## Methods

### Study design and patient selection

This was a retrospective cohort study of consecutive patients with early-stage lung cancer undergoing thoracic surgery at the McGill University Health Centre (MUHC), Montreal, Canada, a large tertiary medical center from September 2017 to June 2021. We included individuals with stage I and II non-small cell lung cancer (NSCLC), treated surgically by lobectomy, segmentectomy, or wedge resection, and with available BEC results within 90 days prior to surgery. We excluded individuals receiving neo-adjuvant therapy, those with small cell lung cancer and carcinoid, as well as patients with bi-lobectomies or pneumonectomies as their postoperative course is often more complex, requiring a prolonged length of stay (LOS) and with a higher risk of complications [25].

### Data source

Data were acquired from the MUHC’s Data Warehouse which contains the cancer registry, hospitalization data, emergency department (ED) visits, International Classification of Diseases − 10th Revision (ICD-10) [26] codes for encounter episodes, laboratory data, imaging metadata, and pharmacy data. We also linked pulmonary function tests (PFTs) done at our institution, where available, which provided information about smoking status. We treated missing data as not missing at random by adding a ‘missing’ for each of the variables derived from that data. This is because patients who are younger and healthy do not routinely get PFTs done as part of their preoperative assessment if they are asymptomatic.

### Outcomes

Our primary outcome was 90-day healthcare utilization, defined as any unscheduled readmission to the index hospital or ED visit within 90 days following surgery. We chose 90-day healthcare utilization as our primary outcome as this is less likely to be affected by surgical technique, surgical volume, and more likely to be impacted by patient-specific factors. Thus, it is more representative of the postoperative recovery [27]. A composite of poor outcomes within 90 days of surgery was defined as readmission to the index hospital or ED visit, death, or prolonged hospital LOS. We defined prolonged hospital LOS as ≥ 7 days during the index admission. We chose to evaluate a composite outcome to capture all the adverse health outcomes that are meanignful for patients given patients may experience the impact of eosinophilia in different ways, some with prolonged hospitalization, some with early death following surgery, and some with increased healthcare utilization after discharge. Secondary outcomes included postoperative pulmonary complications measured as a composite outcome comprising postoperative pneumonia, acute COPD exacerbation, pneumothorax, and prolonged air leak (defined by ICD-10 codes J14, J15.0 to J15.9, J18.1 to J18.9, J85.1, and T81.83) [26]. Prolonged air leak was defined as a persistent air leak lasting more than five days from the day of surgery.

### Exposure of interest and covariates

The main exposure of interest was preoperative BEC within 90 days before the surgery date. BEC has been identified as a biomarker of the eosinophilic type of COPD exacerbations [17] and mainly relates to T helper Th2-driven inflammation [28–30]. BEC was drawn preoperatively and not at a time of hospitalization, ED visit, or suspected acute infection to minimize possible transient causes of eosinophilia. The maximum BEC was considered in cases where more than one result was available for that period. We used a BEC of 200 cells/µL cutoff to categorize the patients into 2 strata (high BEC group versus low BEC group) basing this on the nature of our cohort and previous randomized clinical trials [18, 31, 32]. The BEC threshold of 200 cells/µL is a marker of eosinophilic airway inflammation and response to ICS in patients with COPD exacerbations, with good sensitivity and specificity (90% and 60%, respectively) [17, 33, 34].

Covariates in our models were age, sex, smoking status, Charlson Comorbidity Index (CCI), COPD, asthma, tumor size andnodal status (pathological assessment only)surgical approach (open vs. video-assisted thoracoscopic surgery [VATS])), white blood cells count (WBC), hemoglobin level, and creatinine concentration. Covariates were selected based on clinical significance. CCI was calculated by adding the weights of individual comorbidities on mortality using the ICD-10 codes [35]. COPD was defined by the following ICD-10 codes: J44.0, J44.1, J44.8, and J44.9 [26]. History of asthma was defined by ICD-10 codes beginning with the prefix J45. We used spirometry, where available, to define airway obstruction as an FEV_1_/FVC below 0.70. ICS use was defined as any ICS use, either alone or in combination with a long-acting muscarinic antagonist (LAMA) or long-acting beta-agonist (LABA), identified within 90 days preoperatively or on the day of admission for index surgery from hospital pharmacy records.

### Validation study

To validate our results in a different institution, we performed the same analysis at the Tel-Aviv Medical Center, Israel. Similar inclusion and exclusion criteria were used to identify eligible patients with early-stage NSCLC undergoing thoracic surgery between September 2018 and June 2021. Of note, for the validation study, all medical records were entered with outcomes, complications, and prior diagnoses verified by both ICD-10 codes and physicians’ notes and examinations. Validation was performed for the study primary and secondary outcomes.

### Statistical analysis

We used modified log-Poisson regression with robust variance estimation to examine the factors associated with 90-day healthcare utilization, adjusting for age, sex, smoking status, CCI, COPD and asthma comorbidities, clinical and biological characteristics, and surgical approach. Analyses of the factors associated with 90-day healthcare utilization were also conducted with BECs as a continuous variable. We calculated unadjusted and adjusted relative risks (RR) of 90-day healthcare utilization, death, and prolonged length of stay using a BEC threshold of 200 cells/µL [17, 33]. Analyses were replicated for BEC thresholds of 150 and 300 cells/µL.

A Cox proportional hazards model was used for 1-year survival analysis and adjusted for the same covariates.

A propensity-score weighted analysis was conducted for the main cohort outcomes to balance measured confounding in the groups and strengthen the models’ robustness. The propensity scores were calculated using logistic regression and based on the study covariates, including the types of lung resections (sublobar resections induding wedge resection and segmentectomy; lobar resection: lobectomy).

Inverse probability weighting was applied by taking the inverse of the estimated propensity score.

Sensitivity analysis was conducted with definitions of BEC as follows: (i) the most recent BEC, (ii) the mean of all pre-operative BECs, and (iii) a time-weighted average BEC value. Weights were computed based on the date of surgery with weights ranging from 1 if BEC was assessed 90 days before surgery to 91 for BEC assessed on the day of surgery. E-values were also computed to estimate potential unmeasured counfounding. Lastly, we conducted an exploratory analysis for the 90-day healthcare utilization outcome including an interaction term between BEC and ICS therapy. All analyses were conducted using R Statistical Software Version 4.2.

### Ethics

The study was approved by both institutions’ Research Ethics Boards (2021-6528-MUHC and 0286-24-TLV). Informed consent was waived due to the retrospective study design. The study was conducted in accordance with the Declaration of Helsinki and the Strengthening the Reporting of Observational Studies in Epidemiology (STROBE) checklist.

## Results

### Demographics and clinical characteristics

In total, 715 patients who underwent lobectomy, segmentectomy, or wedge resection for stage I and II NSCLC were included. The clinicodemographic characteristics are described in Table 1.

**Table 1:** Clinical and demographic characteristics of the study population stratified by blood eosinophil count

Variable	Total	BEC < 200	BEC ≥ 200
	*N* = 715	*n* = 569	*n* = 146
Sex, *n* (%)			
Male	299 (41.8%)	229 (40.2%)	70 (48.0%)
Female	416 (58.2%)	340 (59.8%)	76 (52.0%)
Age group, years, n (%)			
< 65	251 (35.1%)	199 (35.0%)	52 (35.6%)
65–79	434 (60.7%)	345 (60.6%)	89 (61.0%)
** ≥** 80	30 (4.2%)	25 (4.4%)	5 (3.4%)
Age, median (IQR)	69 (63–74)	69 (63–73)	69 (63–74)
Smoking history, n (%)			
Current	112 (15.7%)	81 (14.2%)	31 (21.2%)
Former	196 (27.4%)	143 (25.1%)	53 (36.3%)
Never	31 (4.3%)	27 (4.8%)	4 (2.7%)
Missing	376 (52.6%)	318 (55.9%)	58 (39.8%)
CCI, n (%)			
≤ 1	222 (31.0%)	193 (33.9%)	29 (20.0%)
2–3	442 (61.8%)	339 (59.6%)	103 (70.5%)
≥ 4	51 (7.2%)	37 (6.5%)	14 (9.5%)
CCI, Median (IQR)	2 (1–3)	2 (1–2)	2 (2–3)
Obstruction on Spirometry, n (%) (spirometry defined), n (%)			
Yes	110 (15.3%)	77 (13.5%)	33 (22.6%)
No	134 (18.8%)	105 (18.5%)	29 (19.9%)
Missing	471 (65.9%)	386 (68.0%)	84 (57.5%)
COPD (defined by ICD-10)*, n (%)			
Yes	208 (29.0%)	154 (27.0%)	54 (37.0%)
No	507 (71.0%)	415 (73.0%)	92 (63.0%)
Asthma (defined by ICD-10)*, n (%)			
Yes	82 (11.5%)	63 (11.1%)	19 (13.0%)
No	633 (88.5%)	506 (88.9%)	127 (87.0%)
Inhalers, n (%)			
Any ICS	46 (6.4%)	31 (5.4%)	15 (10.2%)
Any LAMA	81 (11.3%)	58 (10.2%)	23 (15.7%)
Any LABA	48 (6.7%)	31 (5.4%)	17 (11.6%)
Tumor size, n (%)			
T1	445 (62.2%)	353 (62.0%)	92 (63.0%)
T2	224 (31.3%)	181 (31.8%)	43 (29.5%)
T3	46 (6.5%)	35 (6.2%)	11 (7.5%)
Nodal status, n (%)			
Nx	37 (5.2%)	32 (5.6%)	5 (3.4%)
N0	617 (86.3%)	491 (86.3%)	126 (86.3%)
N1	60 (8.4%)	45 (8.0%)	15 (10.3%)
Surgical approach, n (%)			
Open	180 (25.2%)	125 (22.0%)	55 (37.7%)
VATS	535 (74.8%)	444 (78.0%)	91 (62.3%)
Resection type, n (%)			
Lobar	480 (67.1%)	380 (66.8%)	100 (68.5%)
Sublobar	235 (32.9%)	189 (33.2%)	46 (31.5%)
Blood results, mean (SD)			
Hemoglobin, g/dL	132.0 (14.8)	131.3 (14.5)	136.3 (15.2)
White blood cells count, cells/µL	9.9 (4.6)	10.1 (4.8)	9.0 (3.0)
Creatinine, µmoles/L	78.6 (28.3)	78.7 (30.2)	78.2 (19.1)

*Definition of abbreviations: BEC* Blood eosinophil count (cells/µL), *CCI* Charlson Comorbidity Index, *ICD* International Classification of Disease, *ICS* Inhaled corticosteroids, *IQR* Interquartile range, *LABA* Long-acting beta-agonist, *LAMA* Long-acting muscarinic antagonist, *SD* Standard Deviation, *VATS* Video-assisted thoracoscopic surgery

*COPD was defined by ICD-10 codes beginning with J44, asthma was defined by ICD-10 codes with the prefix J45

Median (Interquartile Range [IQR]) age was 69 years (63–74), and 41.8% (299/715) were male. Most patients had a moderate comorbidity burden with a CCI of 2–3 (61.8%, 442/715) and 74.8% (535/715) of resections were performed by VATS. Patients with BEC ≥ 200 cells/µL were more likely to be current smokers (21.2% vs. 14.2%, *p* < 0.01), have a COPD diagnosis (37.0% vs. 27.0%) and asthma diagnosis (13.0% vs. 11.1%) based on ICD-10 codes compared with patients with BEC < 200 cells/µL. Moderate comorbidity burden was higher in the high BEC group (70.5% vs. 59.6%, *p* < 0.01). Patients with higher BEC required open thoracotomy more frequently (37.7% vs. 22.0%, *p* = 0.01). The large majority of patients had lobar resections (67.1%, 480/715) with no difference between high and low BEC groups. Patients with BEC ≥ 200 cells/µL were more likely to have obstructive pattern on spirometry (22.6% vs. 13.5%, *p* = 0.01) compared with patients with BEC < 200 cells/µL. The characteristics of the study population stratified by available spirometry status are reported in Appendix Table 1.

### Healthcare utilization

One hundred and ten patients (15.4%, *n* = 110/715) had unscheduled readmissions or ED visits within 90 days following surgery. Patients with BEC ≥ 200 cells/µL had higher rates of ED visits and readmission 19.2% (*n* = 28/146) compared with the low BEC group 14.4% (*n* = 82/569, RR, 1.32, *p* = 0.02). The leading causes of readmission were pulmonary complications occurring in 59.0% (*n* = 65/110) of patients. BEC ≥ 200 cells/µL was associated with a 1.5 times higher risk of healthcare utilization within 90 days of surgery (RR, 1.52; 95% CI, 1.02–2.25) after adjustment (Table 2). In the propensity-score weighted analysis, high BEC ≥ 200 cells/µL remained associated with higher healthcare utilization within 90 days post-surgery (RR, 1.52; 95% CI, 1.02–2.25).

**Table 2:** Factors associated with 90-day healthcare utilization in the study cohort and validation cohort (Log-Poisson regression)

		Study cohort			Validation cohort	
		Unadjusted RR (95% CI)	Adjusted RR (95% CI)	PS weighted RR (95% CI)	Unadjusted RR (95% CI)	Adjusted RR (95% CI)
BEC*, cells/µL	< 200	1 (Ref)	1 (Ref)	1 (Ref)	1 (Ref)	1 (Ref)
	≥ 200	**1.55 (1.07–2.25)**	**1.52 (1.02–2.25)**	**1.52 (1.02–2.25)**	**2.04 (1.09–3.89)**	**2.23 (1.06–4.69)**
Sex	Female	1 (Ref)	1 (Ref)	1 (Ref)	1 (Ref)	1 (Ref)
	Male	1.26 (0.89–1.78)	1.30 (0.89–1.91)	1.33 (0.90–1.95)	0.76 (0.40–1.44)	0.58 (0.27–1.25)
Age, years		1.01 (0.99–1.04)	1.01 (0.99–1.04)	1.01 (0.99–1.04)	1.00 (0.97–1.04)	0.98 (0.93–1.03)
CCI	≤ 1	1 (Ref)	1 (Ref)	1 (Ref)	1 (Ref)	1 (Ref)
	2–3	1.05 (0.71–1.57)	0.84 (0.51–1.36)	0.86 (0.52–1.41)	2.31 (0.28–19.1)	3.11 (0.32–30.6)
	≥ 4	**2.03 (1.17–3.51)**	1.56 (0.78–3.11)	1.63 (0.81–3.27)	6.25 (0.89-49.0)	**12.7 (1.13–143)**
Smoking	Current	1 (Ref)	1 (Ref)	1 (Ref)	1 (Ref)	1 (Ref)
	Former	0.93 (0.38–2.30)	0.82 (0.33–2.03)	0.98 (0.58–1.64)	0.54 (0.27–1.08)	0.47 (0.21–1.07)
	Never	0.96 (0.57–1.61)	0.82 (0.34–1.94)	0.86 (0.54–1.38)	0.48 (0.16–1.42)	0.69 (0.19–2.43)
	Missing	0.84 (0.52–1.36)	0.76 (0.32–1.78)	1.11 (0.45–2.71)	---	---
COPD**		1.49 (1.05–2.11)	1.50 (0.95–2.37)	1.52 (0.96–2.40)	**2.27 (1.14–4.49)**	1.68 (0.77–3.65)
Asthma**		0.93 (0.53–1.62)	1.14 (0.60–2.14)	1.14 (0.60–2.16)	**3.37 (0.87–13.1)**	**4.57 (1.11–22.9)**
Tumor size	T1	1 (Ref)	1 (Ref)	1 (Ref)	1 (Ref)	1 (Ref)
	T2	1.11 (0.77–1.60)	1.10 (0.75–1.62)	1.08 (0.73–1.57)	0.68 (0.31–1.48)	0.56 (0.23–1.37)
	T3	0.90 (0.41–1.95)	0.72 (0.33–1.55)	0.74 (0.34–1.60)	0.40 (0.09–1.82)	0.24 (0.04–1.41)
Nodal status	N0	1 (Ref)	1 (Ref)	1 (Ref)	1 (Ref)	1 (Ref)
	N1	**0.31 (0.10–0.96)**	**0.29 (0.09–0.91)**	**0.29 (0.09–0.92)**	1.99 (0.76–5.22)	1.51 (0.49–4.66)
Surgical Approach	VATS	1 (Ref)	1 (Ref)	1 (Ref)	1 (Ref)	1 (Ref)
	Open	0.97 (0.65–1.45)	0.84 (0.56–1.25)	0.88 (0.59–1.32)	1.21 (0.55–2.67)	1.39 (0.54–3.55)
White blood cells count		1.00 (0.96–1.05)	1.00 (0.95–1.05)	1.00 (0.96–1.05)	0.99 (0.94–1.05)	0.99 (0.91–1.09)
Hemoglobin		1.00 (0.99–1.01)	0.99 (0.98–1.01)	1.00 (0.98–1.01)	0.86 (0.69–1.06)	0.88 (0.68–1.15)
Creatinine		1.00 (0.99-1.00)	0.99 (0.98-1.00)	0.99 (0.98-1.00)	1.12 (0.69–1.82)	0.86 (0.46–1.59)

*Definition of abbreviations: BEC* Blood eosinophil count (cells/µL), *CCI* Charlson Comorbidity Index, *COPD* Chronic Obstructive Pulmonary Disease, *PS* Propensity Score, *RR* Risk ratio, *VATS* Video-assisted thoracoscopic surgery

The RRs for significant variables are in bold text. Adjustments were made for the covariates depicted in the Table. The PS weighted analysis also includes the types of resections, sublobar and lobar

*BEC was defined as the maximal value within 90 days; **COPD was defined by ICD-10 codes with the prefix J44. Asthma was defined by ICD-10 codes with the prefix J45. For the validation cohort, these diagnoses were verified by electronic medical records

Ten patients who died within 90 days of surgery were removed from this analysis leaving 705 patients. When BECs were used as a continuous variable, the risk of readmission per increase of 100 cells/µL in eosinophil count doubled (RR, 2.15; 95% CI, 1.49–3.12, Appendix Table 2) after adjustment. We observed a similar association when natural cubic splines were used for modeling (Appendix Fig. 1): starting from 110 cells/µL, there was an incremental relationship between BEC and the risk of readmission.

Sensitivity analysis of 90-day healthcare utilization with various definitions of BEC are reported in Table 3. Using the maximal BEC value within 90 days, BEC ≥ 200 cells/µL was associated with 90-day healthcare utilization (RR, 1.52; 95% CI, 1.02–2.25), whereas other definitions of BEC were not significant.

**Table 3:** Sensitivity analysis of 90-day healthcare utilization in the main cohort using (A) maximal BEC, (B) most recent BEC, (C) mean of all preoperative BECs, and (D) a time-weighted average BEC within 90 days

		Maximal BEC within 90 days	Most recent BEC	Mean of all preoperative BECs	Time-weighted average BEC
		Adjusted RR (95% CI)	Adjusted RR (95% CI)	Adjusted RR (95% CI)	Adjusted RR (95% CI)
BEC, cells/µL	< 200	1 (Ref)	1 (Ref)	1 (Ref)	1 (Ref)
	≥ 200	**1.52 (1.02–2.25)**	1.44 (0.83–2.50)	1.25 (0.76–2.03)	1.07 (0.62–1.83)
Sex	Female	1 (Ref)	1 (Ref)	1 (Ref)	1 (Ref)
	Male	1.30 (0.89–1.91)	1.28 (0.87–1.91)	1.33 (0.90–1.97)	1.33 (0.90–1.97)
Age, years		1.01 (0.99–1.04)	1.01 (0.99–1.03)	1.01 (0.99–1.04)	1.01 (0.99–1.04)
CCI	≤ 1	1 (Ref)	1 (Ref)	1 (Ref)	1 (Ref)
	2–3	0.84 (0.51–1.36)	0.85 (0.52–1.40)	0.86 (0.52–1.42)	0.86 (0.52–1.42)
	≥ 4	1.56 (0.78–3.11)	1.55 (0.77–3.09)	1.67 (0.84–3.33)	1.67 (0.84–3.33)
Smoking	Current	1 (Ref)	1 (Ref)	1 (Ref)	1 (Ref)
	Former	0.82 (0.33–2.03)	1.06 (0.43–2.59)	1.09 (0.44–2.66)	1.09 (0.44–2.66)
	Never	0.82 (0.34–1.94)	0.94 (0.56–1.58)	0.97 (0.58–1.62)	0.97 (0.58–1.62)0.97 (0.58–1.62)
	Missing	0.76 (0.32–1.78)	0.84 (0.52–1.35)	0.86 (0.54–1.37)	0.86 (0.54–1.37)
COPD*		1.50 (0.95–2.37)	1.53 (0.97–2.42)	1.52 (0.96–2.40)	1.52 (0.96–2.40)
Asthma*		1.14 (0.60–2.14)	1.14 (0.60–2.16)	1.13 (0.60–2.13)	1.13 (0.60–2.13)
Tumor size	T1	1 (Ref)	1 (Ref)	1 (Ref)	1 (Ref)
	T2	1.10 (0.75–1.62)	1.09 (0.74–1.59)	1.08 (0.74–1.58)	1.08 (0.74–1.58)
	T3	0.72 (0.33–1.55)	0.76 (0.35–1.65)	0.74 (0.33–1.65)	0.74 (0.33–1.65)
Nodal status	N0	1 (Ref)	1 (Ref)	1 (Ref)	1 (Ref)
	N1	**0.29 (0.09–0.91)**	**0.29 (0.09–0.93)**	**0.29 (0.09–0.91)**	**0.29 (0.09–0.91)**
Surgical Approach	VATS	1 (Ref)	1 (Ref)	1 (Ref)	1 (Ref)
	Open	0.84 (0.56–1.25)	0.88 (0.59–1.33)	0.88 (0.59–1.32)	0.88 (0.59–1.32)
White blood cells count		1.00 (0.95–1.05)	0.99 (0.95–1.04)	1.00 (0.96–1.04)	1.00 (0.96–1.04)
Hemoglobin		0.99 (0.98–1.01)	1.00 (0.98–1.01)	1.00 (0.99–1.01)	1.00 (0.99–1.01)
Creatinine		0.99 (0.98-1.00)	0.99 (0.98-1.00)	0.99 (0.98-1.00)	0.99 (0.98-1.00)

*Definition of abbreviations: BEC* Blood eosinophil count (cells/µL), *CCI* Charlson Comorbidity Index, *COPD* Chronic Obstructive Pulmonary Disease, *RR* Risk ratio, *VATS* Video-assisted thoracoscopic surgery. *COPD was defined by ICD-10 codes with the prefix J44. Asthma was defined by ICD-10 codes with the prefix J45

Adjustments were made for the covariates depicted in the Table

Significant findings are highlighted in bold

In the exploratory analysis including an interaction term between BEC and ICS therapy, we observed that patients with high BEC ≥ 200 cells/µL who were on ICS therapy, either alone or as part of dual or triple therapy, had lower healthcare utilization within 90 days of surgery (RR, 0.23; 95% CI, 0.05–1.01, *p* for interaction = 0.05, Appendix Table 3).

The median LOS was 3 days (IQR = 2–5). There was a difference in the rates of prolonged LOS between patients with BEC ≥ 200 cells/µL and low BEC (RR, 1.41; 95% CI, 0.97–2.06) after adjustment, even though not statistically significant. VATS surgical approach was associated with a reduced risk of prolonged LOS compared with open surgical approach (RR, 0.35, 95% CI, 0.25–0.49).

Patients with BEC ≥ 150 cells/µL had a 30% higher relative risk (95% CI, 1.02 to 1.62) of the composite outcome of prolonged surgery LOS, acute care readmission or death by postoperative day 90 when compared to patients with BEC < 150 cells/µL. The associations were consistent for each of the composite outcomes (Table 4) and the magnitude of excess risk tended to increase with preoperative BEC thresholds: RR, 1.44 (95% CI, 1.12 to 1.85) for BEC threshold of 200 cells/µL, and RR, 1.81 (95% CI, 1.37 to 2.38) for BEC threshold of 300 cells/µL. The absolute risk difference in the composite outcome was 11% for the BEC threshold of 200 cells/µL (Number Needed to Harm = 9).

**Table 4:** Adverse event risks by preoperative BEC thresholds among 715 patients undergoing lung resection

BEC, cells/µL	No. of patients (%)	Composite: Surgery LOS ≥ 7, ED/hospital readmission, or death by day 90		Surgery LOS ≥ 7 days		ED/hospital readmission by day 90		Death by day 90	
< 150	495 (69%)	135 (27%)	RR 1.30 *p* = 0.037	71 (14%)	RR 1.32	69 (14%)	RR 1.33	7 (1.4%)	RR 1.57
≥ 150	220 (31%)	77 (35%)		42 (19%)		41 (19%)		5 (2.2%)	
< 200	569 (80%)	152 (27%)	RR 1.44 *p* = 0.006	83 (14%)	RR 1.41	82 (14%)	RR 1.32	8 (1.4%)	RR 1.92
≥ 200	146 (20%)	56 (38%)		30 (21%)		28 (19%)		4 (2.7%)	
< 300	647 (90%)	174 (27%)	RR 1.81 *p* < 0.001	93 (14%)	RR 2.02	97 (15%)	RR 1.21	8 (1.2%)	RR 4.83
≥ 300	68 (10%)	33 (49%)		20 (29%)		13 (19%)		4 (5.9%)	

*Definition of abbreviations: BEC* Blood eosinophil count (cells/µL), *ED* Emergency department, *LOS* Length of stay, *RR* Relative Risk

Postoperative adverse event risks and relative risks are summarized at various BEC thresholds

### Complications

There were 130 patients (18.2%) who experienced postoperative pulmonary complications of which 27/146 (18.5%) with BEC ≥ 200 cells/µL compared to 103/569 (18.1%) with no eosinophilia (*p* = 0.54). The most common postoperative complication was prolonged air leak occurring in 38.4% of cases. There was no difference in the risk of postoperative pulmonary complications between patients with high BEC and low BEC after adjustment (RR, 0.86; 95% CI, 0.58–1.27). Similarly, VATS versus open thoracotomy did not impact the risk of postoperative pulmonary complications (RR, 1.09; 95% CI, 0.76–1.56). The presence of COPD, however, was associated with an increased risk of postoperative pulmonary complications (RR, 1.44; 95% CI, 1.00-2.07).

### Mortality

Ten (1.4%) patients died within 90 days of surgery with the earliest death occurring 8 days postoperatively. The rates of 90-day mortality were similar between patients with high BEC compared with those with no eosinophilia (2.7% vs. 1.4%, *p* = 0.10).

All 715 patients had adequate survival data for 1-year survival analysis. Median survival was not reached in the 1 year of follow-up for the entire cohort. Among patients with high BEC, survival probability 1 year following surgery was lower at 92% (95% CI, 88%-96%) compared with 96% (95% CI, 93%-97%) in the low BEC group. A total of 36/715 patients (5.0%) died within 1 year, 11/146 (7.5%) of whom were in the high BEC group compared to 25/569 (4.4%) in the low BEC group. The Kaplan Meier curve of 1-year survival stratified by BEC is reported in Appendix Fig. 2.

In the adjusted Cox proportional hazards model, high BEC ≥ 200 cells/µL was associated with a higher probability of death at 1 year (HR, 2.41; 95% CI, 1.08–5.35) after adjustment (Table 5).

**Table 5:** Cox proportional hazards models for 1-year survival in the study cohort

Variables		Adjusted HR	PS weighted HR
BEC, cells/µL	< 200	1 (Ref)	1 (Ref)
	≥ 200	**2.41 (1.08–5.35)**	**2.76 (1.14–6.67)**
Age at surgery		**1.06 (1.01–1.12)**	**1.11 (1.06–1.17)**
Sex	Female	1 (Ref)	1 (Ref)
	Male	**2.08 (1.01–4.28)**	1.68 (0.83–3.42)
CCI	≤ 1	1 (Ref)	1 (Ref)
	2–3	0.58 (0.21–1.58)	1.35 (0.43–4.21)
	≥ 4	1.28 (0.35–4.68)	2.66 (0.59–11.88)
COPD		**2.51 (1.00-6.30)**	2.21 (0.77–6.37)
Asthma		1.15 (0.28–4.57)	0.56 (0.14–2.27)
Smoking	Current	1 (Ref)	1 (Ref)
	Former	0.49 (0.05–4.72)	1.20 (0.34–4.26)
	Never	0.59 (0.06–5.11)	1.87 (0.62–5.64)
	Missing	0.91 (0.11–7.49)	0.81 (0.05–13.61)
Surgical approach	VATS	1 (Ref)	1 (Ref)
	Open	0.59 (0.25–1.41)	0.66 (0.31–1.38)
Tumor size	T1	1 (Ref)	1 (Ref)
	T2	1.46 (0.64–3.32)	2.08 (0.73–5.94)
	T3	**3.20 (1.24–8.23)**	**5.72 (2.03–16.18)**
Nodal status	N0	1 (Ref)	1 (Ref)
	N1	**2.86 (1.08–7.59)**	**5.03 (1.70-14.92)**
White blood cells count		1.01 (0.96–1.08)	1.00 (0.92–1.09)
Hemoglobin		**0.96 (0.94–0.98)**	**0.96 (0.94–0.97)**
Creatinine		0.99 (0.99-1.00)	0.99 (0.99-1.00)

*Definition of abbreviations: BEC* Blood eosinophil count (cells/µL), *CCI* Charlson Comorbidity Index, *HR* Hazard Ratio, *PS* Propensity Score, *VATS* Video-assisted thoracoscopic surgery

The HRs for significant variables are in bold text. Results are adjusted for age, sex, CCI, COPD, asthma, smoking status, surgical approach, tumor size and nodal status, white blood cells count, hemoglobin, and creatinine. The PS weighted analysis also includes the types of resections, sublobar and lobar

In the propensity-score weighted analysis, high BEC ≥ 200 cells/µL remained associated with higher mortality at 1 year (HR, 2.76; 95% CI, 1.14–6.67).

Other factors associated with poor 1-year survival were male sex, advanced age, increasing tumor size, presence of nodal disease, and COPD comorbidity. Advanced age, tumor size and nodal status remained significant in the propensity-score adjusted analysis. Hemoglobin was inversely associated with poor 1-year survival in both models.

### Validation cohort

There were 234 patients included in the validation cohort (median [IQR] age = 71 years [65–76], 47.4% female). Of them, 47 (20.1%) had unscheduled readmissions or ED visits within 90 days following surgery. Their baseline characteristics are shown in Appendix Table 4. BEC ≥ 200 cells/µL (88 patients, 37.6%) was a predictor for 90-day healthcare utilization in univariate (RR, 2.04; 95% CI, 1.09–3.89) and multivariate (RR, 2.23; 95% CI, 1.06–4.69) analyses (Table 2). BEC as a continuous variable was also associated with 90-day healthcare utilization (RR, 1.42; 95% CI, 1.10–1.94, Appendix Table 5). BEC ≥ 200 cells/µL was not associated with prolonged LOS or with the composite outcome (Appendix Table 6), although LOS was generally longer in the validation group (median [IQR] = 6 [5–8] days). On the contrary, BEC ≥ 300 cells/µL was a risk factor for the composite outcome (RR, 2.45; 95% CI, 1.24–4.84, Appendix Table 6). Both BEC cutoffs of 200 and 300 cells/µL were associated with 90-day mortality. As only 10 patients died during the 1-year following surgery, further analyses regarding this outcome were inadequate.

## Discussion

This is the first study to report associations between high BECs and postoperative outcomes and survival in early-stage lung cancer patients presenting for pulmonary resection, with higher healthcare utilization and lower 1-year survival. The readmission rate of 15% in our study was consistent with that reported in other studies which showed rates between 7% and 23%, with most studies having 90-day readmission rates above 18% [27, 36]. Those who were readmitted had a lower overall survival consistent with previous studies [37, 38].To our knowledge, this is the first study that has explored BEC as a predictor of surgical outcomes and mortality among patients with early-stage lung cancer. Since the paucity of data about the significance of BECs in early-stage lung cancer, this study provides an interesting intersection for two diseases that often coexist, lung cancer and obstructive airway disease. Lung resection remains a high-risk procedure and despite the many advances in surgical technique and postoperative care, mortality and readmission rates remain high [25, 39]. Our results demonstrate the associations between eosinophilia and poor surgical outcomes in the entire cohort among patients with or without respiratory diseases. Of note, nodal status N1 was associated with lower 90-day healthcare utilization compared to N0. This was only observed in the MUHC cohort and not in the validation cohort, therefore cautious is needed when drawing conclusions. One hypothesis is that patients with N1 positivity may reflect a more extensive lymph node dissection by more experienced surgeons, reducing the rate of post operative complications and health care utilization.

The thoracic surgery literature has focused on immediate postoperative complications within 30 days rather than 90 days [40, 41]. However, we hypothesize that as the time since surgery increases, the complication rates and readmissions are more reflective of the patient’s baseline health status and comorbidities than surgical technique. Readmissions after lung cancer resection surgery have been associated with an increase in mortality [37], similar to the association with COPD readmissions and mortality [42]. Risk of death increased 30-40-fold within the first 3 months after a COPD admission, with the adjusted hazard ratio of death increasing from 1.0 to 5.2 (95% CI, 4.9–5.5) from the first to the tenth readmission [42]. It is difficult to ignore this competing risk on mortality for early-stage lung cancer presenting for resection who may have confirmed or potentially undiagnosed airway disease.

Postoperative pulmonary complications contribute to 25–55% of readmissions following lobectomy [40, 43]. Consistent with other studies, the commonest causes of unplanned readmissions in the current study were pulmonary, mainly pneumonia, empyema, and pneumothorax. Post-operative pulmonary complications showed no difference between patients with high and low BEC. We hypothesize that blood-based inflammatory markers may preferentially influence systemic outcomes such as readmission and mortality rather than organ-specific pulmonary outcomes. Peri-operative systemic inflammation—potentially reflecting eosinophil-driven airway disease or unrecognized COPD/asthma exacerbations—can destabilize atherosclerotic plaques by promoting endothelial dysfunction and fibrous cap weakening. Surgical stress may act as a second hit, triggering cardiovascular events without increasing pulmonary complications. This is consistent with evidence that COPD exacerbations markedly increase short- and long-term mortality, largely through cardiovascular mechanisms rather than respiratory failure [44–46], suggesting that blood-based inflammation captures systemic vascular risk not reflected in post-operative pulmonary complications rates. Furthermore, open thoracotomy was not associated with a higher risk of readmission compared to VATS consistent with other studies [40, 47]. However, we did not observe association between surgical approach and postoperative pulmonary complications, as reported by Bhagat et al. [40]. These differences could be explained by the differences in the study population since we only included early-stage lung cancer and excluded bi-lobectomies and pneumonectomies.

Some studies have found benefit in separating the pulmonary complications instead of using them as a composite variable [43]. For example, complications like pulmonary embolus and empyema had a greater association with readmissions than prolonged air leak for example [43]. Given our sample characteristics, we found that using the composite variable for pulmonary complications was more feasible and less prone to misclassification based on ICD-10 codes. Recently much of the surgical literature has focused on open versus VATS technique to reduce postoperative complications [48]. Less focus has been made on the management of comorbidities such as obstructive airway disease, which may have an equally important contribution to mortality.

In COPD, BECs have been identified as an important actionable biomarker for improved disease control and mortality [49]. BECs ≥ 200 cells/µL were found to be the strongest predictor of reduction in all-cause mortality among patients with COPD who are on more than 6 months of inhaled ICS therapy [49]. Further investigation into the role of eosinophilia in all patients presenting for lung resection, regardless of known airway disease, may then be warranted.

In our study, we found an association between ICS use in the preoperative period and lower healthcare utilization for patients with high BECs. This trend indicates a potential benefit of ICS treatment in this population. Although this analysis remains exploratory given the small numbers of patients with high BEC and on ICS (*n* = 15) and the observational study design that do not allow conclusion on causality. Few studies explored the use of inhaler therapy that includes ICS (LAMA/LABA and triple therapy combinations) for COPD prescribed a few weeks before lung cancer surgery continuing for up to 3 months with promising results [50, 51]. Given the substantial evidence about the improvement of clinical symptoms in patients with type 2 inflammation COPD who are put on ICS therapy [31, 52], there may be a benefit in exploring ICS or biologic anti-IL5 use among all patients who have high BECs in the preoperative surgical period. Prospective evaluations of the safety of those therapeutic options with respect to wound healing and infection risk is also required.

Regarding the translational potential, BEC is a recognized proxy of eosinophilic airway inflammation [53] that is increasingly used for targeted therapy with anti-IL5 treatment in asthma and COPD [15]. Our results indicate that BEC represents a simple and actionable biomarker for risk stratification in lung resection. Existing preoperative tools, such as ASA physical status assessment, have poor performance in current lung cancer patients as they were developed in all thoracic surgery and on older cohorts with a larger proportion of open thoracotomy and lesser representation of minimally invasive surgery [54]. The Revised Cardiac Risk Index (RCRI) and Thoracic Revised Cardiac Risk Revised Cardiac Risk Index (ThRCRI) scores poorly perform to accurately predict the risk of cardiac complications in patients undergoing resection of lung cancer [55]. The Thoracic Surgery Scoring System (Thoracoscore) has shown poor discriminative and predictive ability for mortality and postoperative complications following lung resection [56].

### Limitations

First limitation of this study is the retrospective study design. However, data originated from the MUHC Data Warehouse that centralizes healthcare data from clinical and administrative systems strengthening data consistency. The E-value for the BEC and 90-day outcome point estimate was 2.41 (meaning that residual confounding could explain the association if an unmeasured covariate has a risk association of at least 2.41) and 1.20 for the confidence interval bound closest to the null suggesting the association is moderately robust to unmeasured confounding [57]. Our results were also validated by a second cohort and the rates of adverse clinical outcomes we report while in line with published rates, are consistent with high-volume surgical centers such as those included. Second, BECs used were taken at different time points before surgery for participants and 53% of the participants in the main cohort had at least 2 BECs measures. Variability in BECs has been well studied and there is a suggestion that there may be a diurnal as well as seasonal variations [58, 59]. In one study, up to 22% of patients with COPD had important variability in their BECs at 1 year follow-up [60]. To mitigate this limitation and reinforce the robustness of our models, we conducted a sensitivity analysis with several definitions of BEC in the 90-day preoperative window. Because BECs were collected for clinical reasons at variable timepoints in our study, future trials should include standardized sampling intervals in longitudinal follow-ups. Third, this study did not exclusively focus on patients with a diagnosis of COPD or asthma, as this was defined with ICD-10 codes, however the burden of undiagnosed and untreated asthma and COPD is known to be substantial in lung cancer screening and lung cancer populations [6, 7], making these results more generalizable. Though we did look at spirometry data for obstruction, some of the many pulmonary function tests were performed outside our institution and were not available for the analysis. While it does limit the interpretation of our results, the association between BECs and postoperative healthcare utilization at 90 days remained significant after adjustment for COPD or asthma from ICD-10 codes, suggesting that BECs are a biomarker independent of the presence of airway diseases. In the CanCOLD cohort, high BECs ≥ 300 cells/µL were associated with a more rapid decline in FEV_1_ in the presence or absence of COPD [20]. This suggests that there may be biologic relevance to examining eosinophilia in all patients presenting for resection of early-stage lung cancer, regarless of the presence of airway diseases. Fourth, excluding bi-lobectomy and pneumonectomy limits the generalizability. Lastly, the validation cohort was relatively small and several outcomes were not replicated (e.g., prolonged surgical LOS and mortality), undermining the external validity.Surgical LOS was not assessed because of different practice patterns and reimbursements postoperatively in the two countries, the median LOS being of 3 days in the main cohort and 6 days in the validation cohort. This research is hypothesis-generating and should not guide peri-operative prescribing outside clinical trials. To support our findings, future research should prioritize prospective studies that broadly explore potential factors influencing eosinophilia, thereby ensuring validation of our conclusions.

## Conclusions

We found that high preoperative BECs were associated with poor surgical outcomes after lung cancer resection. This highlights that blood eosinophils may be an inexpensive, readily available and important biomarker for both readmission and mortality in all individuals presenting for early-stage lung cancer resection. We hope those findings will help improve postoperative outcomes. Further study should notably identify the role of combined ICS and bronchodilator therapy, and potentially biologic therapy to improve postoperative outcomes in this population.

## Supplementary Information

Supplementary Material 1.

## Data Availability

The datasets supporting the conclusions of this article are available from the corresponding author on reasonable request.

## Abbreviations

BEC: Blood eosinophil count
CCI: Charlson Comorbidity Index
CI: Confidence Interval
COPD: Chronic obstructive pulmonary disease
ED: Emergency department
FEV_1_: Forced Expiratory Volume in 1 s
HR: Hazard Ratio
ICD-10: International Classification of Disease, 10th Revision
ICS: Inhaled Corticosteroids
IQR: Interquartile Range
LABA: Long-acting beta-agonist
LAMA: Long-acting muscarinic antagonist
LOS: Length of stay
MUHC: McGill University Health Centre
NSCLC: Non-Small Cell Lung Cancer
PFT: Pulmonary Function Test
RR: Relative Risk
VATS: Video-assisted thoracoscopic surgery

## References

[1] Li C, Wang H, Jiang Y, Fu W, Liu X, Zhong R, et al. Advances in lung cancer screening and early detection. Cancer Biol Med. 2022;19(5):591–608. 35535966 10.20892/j.issn.2095-3941.2021.0690 PMC9196057

[2] Gajra A, Akbar SA, Din NU. Management of Lung Cancer in the Elderly. Clin Geriatr Med. 2016;32(1):81–95. 26614862 10.1016/j.cger.2015.08.008

[3] Loganathan RS, Stover DE, Shi W, Venkatraman E. Prevalence of COPD in women compared to men around the time of diagnosis of primary lung cancer. Chest. 2006;129(5):1305–12. 16685023 10.1378/chest.129.5.1305

[4] Young RP, Hopkins RJ, Christmas T, Black PN, Metcalf P, Gamble GD. COPD prevalence is increased in lung cancer, independent of age, sex and smoking history. Eur Respir J. 2009;34(2):380–6. 19196816 10.1183/09031936.00144208

[5] Roy E, Rheault J, Pigeon MA, Ugalde PA, Racine C, Simard S, et al. Lung cancer resection and postoperative outcomes in COPD: A single-center experience. Chron Respir Dis. 2020;17:1479973120925430. 32468842 10.1177/1479973120925430 PMC7263105

[6] Mouronte-Roibás C, Leiro-Fernández V, Ruano-Raviña A, Ramos-Hernández C, Abal-Arca J, Parente-Lamelas I, et al. Chronic Obstructive Pulmonary Disease in Lung Cancer Patients: Prevalence, Underdiagnosis, and Clinical Characterization. Respir Int Rev Thorac Dis. 2018;95(6):414–21. 10.1159/000487243 29587299

[7] Ruparel M, Quaife SL, Dickson JL, Horst C, Tisi S, Hall H, et al. Prevalence, Symptom Burden, and Underdiagnosis of Chronic Obstructive Pulmonary Disease in a Lung Cancer Screening Cohort. Ann Am Thorac Soc. 2020;17(7):869–78. 32164439 10.1513/AnnalsATS.201911-857OC PMC7328177

[8] García-Pardo M, Chang A, Schmid S, Dong M, Brown MC, Christiani D, et al. Respiratory and Cardiometabolic Comorbidities and Stages I to III NSCLC Survival: A Pooled Analysis From the International Lung Cancer Consortium. J Thorac Oncol Off Publ Int Assoc Study Lung Cancer. 2023;18(3):313–23. 10.1016/j.jtho.2022.10.020 PMC10463560 36396063

[9] Goffin JR, Corriveau S, Tang GH, Pond GR. Management and outcomes of patients with chronic obstructive lung disease and lung cancer in a public healthcare system. PLoS ONE. 2021;16(5):e0251886. 33999942 10.1371/journal.pone.0251886 PMC8128239

[10] Islam KMM, Jiang X, Anggondowati T, Lin G, Ganti AK. Comorbidity and Survival in Lung Cancer Patients. Cancer Epidemiol Biomark Prev Publ Am Assoc Cancer Res Cosponsored Am Soc Prev Oncol. 2015;24(7):1079–85. 10.1158/1055-9965.EPI-15-0036 26065838

[11] Perrotta F, D’Agnano V, Scialò F, Komici K, Allocca V, Nucera F, et al. Evolving concepts in COPD and lung cancer: a narrative review. Minerva Med. 2022;113(3):436–48. 35156786 10.23736/S0026-4806.22.07962-9

[12] Agustí A, Celli BR, Criner GJ, Halpin D, Anzueto A, Barnes P, et al. Global Initiative for Chronic Obstructive Lung Disease 2023 Report: GOLD Executive Summary. Eur Respir J. 2023;61(4):2300239. 36858443 10.1183/13993003.00239-2023 PMC10066569

[13] Whittaker H, Rubino A, Müllerová H, Morris T, Varghese P, Xu Y, et al. Frequency and Severity of Exacerbations of COPD Associated with Future Risk of Exacerbations and Mortality: A UK Routine Health Care Data Study. Int J Chron Obstruct Pulmon Dis. 2022;17:427–37. 35264849 10.2147/COPD.S346591 PMC8901192

[14] Slenter RHJ, Sprooten RTM, Kotz D, Wesseling G, Wouters EFM, Rohde GGU. Predictors of 1-year mortality at hospital admission for acute exacerbations of chronic obstructive pulmonary disease. Respir Int Rev Thorac Dis. 2013;85(1):15–26. 10.1159/000342036 23037178

[15] Ramakrishnan S, Russell REK, Mahmood HR, Krassowska K, Melhorn J, Mwasuku C, et al. Treating eosinophilic exacerbations of asthma and COPD with benralizumab (ABRA): a double-blind, double-dummy, active placebo-controlled randomised trial. Lancet Respir Med. 2025;13(1):59–68. 39615502 10.1016/S2213-2600(24)00299-6

[16] Bathoorn E, Liesker JJW, Postma DS, Koëter GH, van der Toorn M, van der Heide S, et al. Change in inflammation in out-patient COPD patients from stable phase to a subsequent exacerbation. Int J Chron Obstruct Pulmon Dis. 2009;4:101–9. 19436694 10.2147/copd.s4854 PMC2672798

[17] Bafadhel M, McKenna S, Terry S, Mistry V, Reid C, Haldar P, et al. Acute exacerbations of chronic obstructive pulmonary disease: identification of biologic clusters and their biomarkers. Am J Respir Crit Care Med. 2011;184(6):662–71. 21680942 10.1164/rccm.201104-0597OC

[18] Harries TH, Rowland V, Corrigan CJ, Marshall IJ, McDonnell L, Prasad V, et al. Blood eosinophil count, a marker of inhaled corticosteroid effectiveness in preventing COPD exacerbations in post-hoc RCT and observational studies: systematic review and meta-analysis. Respir Res. 2020;21(1):3. 31900184 10.1186/s12931-019-1268-7 PMC6942335

[19] Bafadhel M, McKenna S, Terry S, Mistry V, Pancholi M, Venge P, et al. Blood Eosinophils to Direct Corticosteroid Treatment of Exacerbations of Chronic Obstructive Pulmonary Disease. Am J Respir Crit Care Med. 2012;186(1):48–55. 22447964 10.1164/rccm.201108-1553OC PMC3400995

[20] Tan WC, Bourbeau J, Nadeau G, Wang W, Barnes N, Landis SH, et al. High eosinophil counts predict decline in FEV1: results from the CanCOLD study. Eur Respir J. 2021;57(5):2000838. 33303555 10.1183/13993003.00838-2020

[21] Lipson DA, Barnhart F, Brealey N, Brooks J, Criner GJ, Day NC, et al. Once-Daily Single-Inhaler Triple versus Dual Therapy in Patients with COPD. N Engl J Med. 2018;378(18):1671–80. 29668352 10.1056/NEJMoa1713901

[22] Martinez FJ, Rabe KF, Ferguson GT, Wedzicha JA, Singh D, Wang C, et al. Reduced All-Cause Mortality in the ETHOS Trial of Budesonide/Glycopyrrolate/Formoterol for Chronic Obstructive Pulmonary Disease. A Randomized, Double-Blind, Multicenter, Parallel-Group Study. Am J Respir Crit Care Med. 2021;203(5):553–64. 33252985 10.1164/rccm.202006-2618OC PMC7924571

[23] Sibille A, Corhay JL, Louis R, Ninane V, Jerusalem G, Duysinx B. Eosinophils and Lung Cancer: From Bench to Bedside. Int J Mol Sci. 2022;23(9):5066. 35563461 10.3390/ijms23095066 PMC9101877

[24] Kaes J, Van der Borght E, Vanstapel A, Van Herck A, Sacreas A, Heigl T, et al. Peripheral Blood Eosinophilia Is Associated with Poor Outcome Post-Lung Transplantation. Cells. 2020;9(11):2516. 33233857 10.3390/cells9112516 PMC7699939

[25] Rotman JA, Plodkowski AJ, Hayes SA, de Groot PM, Shepard JAO, Munden RF, et al. Postoperative complications after thoracic surgery for lung cancer. Clin Imaging. 2015;39(5):735–49. 26117564 10.1016/j.clinimag.2015.05.013

[26] World Health Organization. ICD-10: International statistical classification of diseases and related health problems : tenth revision. 2nd ed 2004.

[27] Konstantinidis K, Woodcock-Shaw J, Dinesh P, Brunelli A. Incidence and risk factors for 90-day hospital readmission following video-assisted thoracoscopic anatomical lung resection†. Eur J Cardio-Thorac Surg Off J Eur Assoc Cardio-Thorac Surg. 2019;55(4):666–72. 10.1093/ejcts/ezy345 30364954

[28] Folci M, Ramponi G, Arcari I, Zumbo A, Brunetta E. Eosinophils as Major Player in Type 2 Inflammation: Autoimmunity and Beyond. Adv Exp Med Biol. 2021;1347:197–219. 34031864 10.1007/5584_2021_640

[29] Gopallawa I, Dehinwal R, Bhatia V, Gujar V, Chirmule N. A four-part guide to lung immunology: Invasion, inflammation, immunity, and intervention. Front Immunol. 2023;14:1119564. 37063828 10.3389/fimmu.2023.1119564 PMC10102582

[30] Moldoveanu B, Otmishi P, Jani P, Walker J, Sarmiento X, Guardiola J, et al. Inflammatory mechanisms in the lung. J Inflamm Res. 2009;2:1–11. 22096348 PMC3218724

[31] Hastie AT, Martinez FJ, Curtis JL, Doerschuk CM, Hansel NN, Christenson S, et al. Association of sputum and blood eosinophil concentrations with clinical measures of COPD severity: an analysis of the SPIROMICS cohort. Lancet Respir Med. 2017;5(12):956–67. 29146301 10.1016/S2213-2600(17)30432-0 PMC5849066

[32] Bafadhel M, Greening NJ, Harvey-Dunstan TC, Williams JEA, Morgan MD, Brightling CE, et al. Blood Eosinophils and Outcomes in Severe Hospitalized Exacerbations of COPD. Chest. 2016;150(2):320–8. 26851799 10.1016/j.chest.2016.01.026

[33] Negewo NA, McDonald VM, Baines KJ, Wark PA, Simpson JL, Jones PW, et al. Peripheral blood eosinophils: a surrogate marker for airway eosinophilia in stable COPD. Int J Chron Obstruct Pulmon Dis. 2016;11:1495–504. 27445469 10.2147/COPD.S100338 PMC4936821

[34] Bafadhel M, Davies L, Calverley PMA, Aaron SD, Brightling CE, Pavord ID. Blood eosinophil guided prednisolone therapy for exacerbations of COPD: a further analysis. Eur Respir J. 2014;44(3):789–91. 24925917 10.1183/09031936.00062614

[35] Charlson ME, Pompei P, Ales KL, MacKenzie CR. A new method of classifying prognostic comorbidity in longitudinal studies: development and validation. J Chronic Dis. 1987;40(5):373–83. 3558716 10.1016/0021-9681(87)90171-8

[36] García-Tirado J, Júdez-Legaristi D, Landa-Oviedo HS, Miguelena-Bobadilla JM. Unplanned readmission after lung resection surgery: A systematic review. Cir Esp. 2019;97(3):128–44. 30545643 10.1016/j.ciresp.2018.11.005

[37] Hu Y, McMurry TL, Isbell JM, Stukenborg GJ, Kozower BD. Readmission after lung cancer resection is associated with a 6-fold increase in 90-day postoperative mortality. J Thorac Cardiovasc Surg. 2014;148(5):2261–e22671. 24823283 10.1016/j.jtcvs.2014.04.026 PMC4201876

[38] Hu Y, McMurry TL, Stukenborg GJ, Kozower BD. Readmission predicts 90-day mortality after esophagectomy: Analysis of Surveillance, Epidemiology, and End Results Registry linked to Medicare outcomes. J Thorac Cardiovasc Surg. 2015;150(5):1254–60. 26412319 10.1016/j.jtcvs.2015.08.071 PMC4637190

[39] Granger C, Cavalheri V. Preoperative exercise training for people with non-small cell lung cancer. Cochrane Database Syst Rev. 2022;9(9):CD012020. 36170564 10.1002/14651858.CD012020.pub3 PMC9519181

[40] Bhagat R, Bronsert MR, Ward AN, Martin J, Juarez-Colunga E, Glebova NO, et al. National Analysis of Unplanned Readmissions After Thoracoscopic Versus Open Lung Cancer Resection. Ann Thorac Surg. 2017;104(6):1782–90. 29102302 10.1016/j.athoracsur.2017.08.047

[41] Hendriksen BS, Reed MF, Taylor MD, Hollenbeak CS. Readmissions After Lobectomy in an Era of Increasing Minimally Invasive Surgery: A Statewide Analysis. Innov Phila Pa. 2019;14(5):453–62. 10.1177/1556984519874064 31533516

[42] Suissa S, Dell’Aniello S, Ernst P. Long-term natural history of chronic obstructive pulmonary disease: severe exacerbations and mortality. Thorax. 2012;67(11):957–63. 22684094 10.1136/thoraxjnl-2011-201518 PMC3505864

[43] Stiles BM, Poon A, Giambrone GP, Gaber-Baylis LK, Wu X, Lee PC, et al. Incidence and Factors Associated With Hospital Readmission After Pulmonary Lobectomy. Ann Thorac Surg. 2016;101(2):434–42. diacussion 442–443. 26718860 10.1016/j.athoracsur.2015.10.001

[44] Aaron SD. COPD Exacerbations and Future Risk of Serious Cardiovascular Events. Chest. 2024;166(6):1262–3. 39663021 10.1016/j.chest.2024.08.036

[45] Wallström O, Stridsman C, Lindberg A, Nyberg F, Vanfleteren LEGW. Exacerbation History and Risk of Myocardial Infarction and Pulmonary Embolism in COPD. Chest. 2024;166(6):1347–59. 39094732 10.1016/j.chest.2024.07.150 PMC11638550

[46] Kunisaki KM, Dransfield MT, Anderson JA, Brook RD, Calverley PMA, Celli BR, et al. Exacerbations of Chronic Obstructive Pulmonary Disease and Cardiac Events. A Post Hoc Cohort Analysis from the SUMMIT Randomized Clinical Trial. Am J Respir Crit Care Med. 2018;198(1):51–7. 29442524 10.1164/rccm.201711-2239OC PMC6913068

[47] Assi R, Wong DJ, Boffa DJ, Detterbeck FC, Wang Z, Chupp GL, et al. Hospital readmission after pulmonary lobectomy is not affected by surgical approach. Ann Thorac Surg. 2015;99(2):393–8. 25497070 10.1016/j.athoracsur.2014.10.014

[48] Klapper J, D’Amico TA. VATS versus open surgery for lung cancer resection: moving toward a minimally invasive approach. J Natl Compr Cancer Netw JNCCN. 2015;13(2):162–4. 25691607 10.6004/jnccn.2015.0023

[49] Chen H, Deng ZX, Sun J, Huang Q, Huang L, He YH, et al. Association of Inhaled Corticosteroids With All-Cause Mortality Risk in Patients With COPD: A Meta-analysis of 60 Randomized Controlled Trials. Chest. 2023;163(1):100–14. 35921883 10.1016/j.chest.2022.07.015

[50] Bölükbas S, Eberlein M, Eckhoff J, Schirren J. Short-term effects of inhalative tiotropium/formoterol/budenoside versus tiotropium/formoterol in patients with newly diagnosed chronic obstructive pulmonary disease requiring surgery for lung cancer: a prospective randomized trial. Eur J Cardio-Thorac Surg Off J Eur Assoc Cardio-Thorac Surg. 2011;39(6):995–1000. 10.1016/j.ejcts.2010.09.025 20970351

[51] Homma T. Preoperative umeclidinium/vilanterol or tiotropium improves postoperative FEV1 in lung cancer patients with comorbid untreated chronic obstructive pulmonary disease. J Thorac Dis. 2023;15(4):1584–94. 37197513 10.21037/jtd-22-1704 PMC10183516

[52] Pascoe S, Barnes N, Brusselle G, Compton C, Criner GJ, Dransfield MT, et al. Blood eosinophils and treatment response with triple and dual combination therapy in chronic obstructive pulmonary disease: analysis of the IMPACT trial. Lancet Respir Med. 2019;7(9):745–56. 31281061 10.1016/S2213-2600(19)30190-0

[53] Bafadhel M, Pavord ID, Russell REK. Eosinophils in COPD: just another biomarker? Lancet Respir Med. 2017;5(9):747–59. 28601554 10.1016/S2213-2600(17)30217-5

[54] Ghatak A. Improving lung cancer outcomes: risk prediction of postoperative readmission and assessing the efficacy of smoking cessation interventions. [Internet]. McGill; 2022. Available from: https://escholarship.mcgill.ca/concern/theses/bn999c83d.

[55] Wotton R, Marshall A, Kerr A, Bishay E, Kalkat M, Rajesh P, et al. Does the revised cardiac risk index predict cardiac complications following elective lung resection? J Cardiothorac Surg. 2013;8:220. 24289748 10.1186/1749-8090-8-220 PMC3879030

[56] Bradley A, Marshall A, Abdelaziz M, Hussain K, Agostini P, Bishay E, et al. Thoracoscore fails to predict complications following elective lung resection. Eur Respir J. 2012;40(6):1496–501. 22496319 10.1183/09031936.00218111

[57] VanderWeele TJ, Ding P. Sensitivity Analysis in Observational Research: Introducing the E-Value. Ann Intern Med. 2017;167(4):268–74. 28693043 10.7326/M16-2607

[58] Benson VS, Hartl S, Barnes N, Galwey N, Van Dyke MK, Kwon N. Blood eosinophil counts in the general population and airways disease: a comprehensive review and meta-analysis. Eur Respir J. 2022;59(1):2004590. 34172466 10.1183/13993003.04590-2020 PMC8756293

[59] Chipps BE, Jarjour N, Calhoun WJ, Iqbal A, Haselkorn T, Yang M, et al. A Comprehensive Analysis of the Stability of Blood Eosinophil Levels. Ann Am Thorac Soc. 2021;18(12):1978–87. 33891831 10.1513/AnnalsATS.202010-1249OC PMC8641810

[60] Yoon JK, Lee JK, Lee CH, Hwang YI, Kim H, Park D, et al. The Association Between Eosinophil Variability Patterns and the Efficacy of Inhaled Corticosteroids in Stable COPD Patients. Int J Chron Obstruct Pulmon Dis. 2020;15:2061–70. 32943859 10.2147/COPD.S258353 PMC7473991

