# Supplementary material for: Blood eosinophil counts and postoperative outcomes in early-stage lung cancer: a retrospective cohort study
Source: Respir Res. 2026 May 1;27:255. doi: 10.1186/s12931-026-03672-9 (PMC13289531; doi:10.1186/s12931-026-03672-9)

| **Appendix** **Table 1.** Study population stratified by available spirometry status | | | |
| --- | --- | --- | --- |
| Variables | Total | Not Missing | Missing |
|  | N = 715 | n = 244 | n = 471 |
| BEC, n (%) |  |  |  |
| < 200 | 569 (79.6%) | 182 (74.6%) | 387 (82.2%) |
| ≥ 200 | 146 (20.4%) | 62 (25.4%) | 84 (17.8%) |
| Sex, n (%) |  |  |  |
| Male | 299 (41.8%) | 102 (41.8%) | 197 (41.8%) |
| Female | 416 (58.2%) | 142 (58.2%) | 274 (58.2%) |
| Age group, years, n (%) |  |  |  |
| <65 | 252 (35.2%) | 83 (34.0%) | 169 (35.9%) |
| 65-79 | 434 (60.7%) | 152 (62.3%) | 282 (59.9%) |
| ≥80 | 29 (4.1%) | 9 (3.7%) | 20 (4.2%) |
| Age, median (IQR) | 69 (63-74) | 70 (63-75) | 68 (63-73) |
| Smoking history, n (%) |  |  |  |
| Current | 112 (15.7%) | 56 (23.0%) | 56 (11.9%) |
| Former | 196 (27.4%) | 141 (57.8%) | 55 (11.7%) |
| Never | 31 (4.3%) | 31 (12.7%) | 0 (0.0%) |
| Missing | 376 (52.6%) | 16 (6.6%) | 360 (76.4%) |
| CCI, n (%) |  |  |  |
| ≤1 | 222 (31.0%) | 68 (27.9%) | 154 (32.7%) |
| 2-3 | 442 (61.8%) | 154 (63.1%) | 288 (61.1%) |
| ≥4 | 51 (7.1%) | 22 (9.0%) | 29 (6.2%) |
| CCI, Median (IQR) | 2 (1-3) | 2 (1-3) | 2 (1-2) |
| Obstruction on Spirometry, n (%) |  |  |  |
| Yes | 110 (15.4%) | 110 (45.1%) | 0 (0.0%) |
| No | 134 (18.7%) | 134 (54.9%) | 0 (0.0%) |
| COPD (defined by ICD-10)*, n (%) |  |  |  |
| Yes | 208 (29.1%) | 70 (28.7%) | 138 (29.3%) |
| No | 507 (70.9%) | 174 (71.3%) | 333 (70.7%) |
| Asthma (defined by ICD-10)*, n (%) |  |  |  |
| Yes | 82 (11.5%) | 36 (14.8%) | 46 (9.8%) |
| No | 633 (88.5%) | 208 (85.2%) | 425 (90.2%) |
| Inhalers, n (%) |  |  |  |
| Any ICS | 46 (6.4%) | 16 (6.6%) | 30 (6.4%) |
| Any LAMA | 81 (11.3%) | 28 (11.5%) | 53 (11.3%) |
| Any LABA | 48 (6.7%) | 19 (7.8%) | 29 (6.2%) |
| Tumor size, n (%) |  |  |  |
| T1 | 445 (62.2%) | 159 (65.2%) | 286 (60.7%) |
| T2 | 224 (31.3%) | 73 (29.9%) | 151 (32.1%) |
| T3 | 46 (6.4%) | 12 (4.9%) | 34 (7.2%) |
| Nodal status, n (%) |  |  |  |
| NX | 37 (5.2%) | 22 (9.0%) | 15 (3.2%) |
| N0 | 617 (86.3%) | 206 (84.4%) | 411 (87.3%) |
| N1 | 60 (8.4%) | 16 (6.6%) | 44 (9.3%) |
| Surgical approach, n (%) |  |  |  |
| Open | 180 (25.2%) | 87 (35.7%) | 93 (19.7%) |
| VATS | 535 (74.8%) | 157 (64.3%) | 378 (80.3%) |
| Resection type, n (%) |  |  |  |
| Lobar | 480 (67.1%) | 160 (65.6%) | 320 (67.9%) |
| Sublobar | 235 (32.9%) | 84 (34.4%) | 151 (32.1%) |
| Blood results, mean (SD) |  |  |  |
| Hemoglobin, g/dL | 132.3 (14.8) | 133.3 (14.3) | 131.8 (15.0) |
| White blood cells count, cells/µL | 9.9 (4.6) | 8.6 (3.4) | 10.5 (5.0) |
| Creatinine, µmoles/L | 78.6 (28.3) | 78.8 (21.9) | 78.5 (31.1) |

## *Definition of abbreviations:* BEC = Blood eosinophil count (cells/µL); CCI = Charlson Comorbidity Index; ICD = International Classification of Disease; ICS = Inhaled corticosteroids; IQR = Interquartile range; LABA = Long-acting beta-agonist; LAMA = Long-acting muscarinic antagonist; SD = Standard Deviation; VATS = Video-assisted thoracoscopic surgery.

## *COPD was defined by ICD-10 codes beginning with J44, asthma was defined by ICD-10 codes with the prefix J45.

## **Appendix Table 2:** Factors independently associated with 90-day healthcare utilization in the study cohort

| Variable | | Unadjusted Risk Ratio  (95% CI) | p-value | Adjusted Risk Ratio  (95% CI) | p-value |
| --- | --- | --- | --- | --- | --- |
| **BEC, cells/µL*** | | **2.04 (1.60-2.60)** | **<0.0001** | **2.15 (1.49-3.12)** | **<0.0001** |
| **Charlson comorbidity index*** | | **1.23 (1.10-1.39)** | **<0.01** | **1.23 (1.06-1.42)** | **<0.001** |
| **Nodal status** | |  |  |  |  |
|  | **N0** | 1 (Ref) | - | 1 (Ref) | - |
|  | **N1** | **0.31 (0.10-0.95)** | **0.04** | **0.28 (0.09-0.90)** | **0.02** |

*Definition of abbreviations*: BEC = Blood eosinophil count (cells/µL); CI = Confidence Interval.

*BEC and the Charlson comorbidity index were used as continuous variables. Adjusted for age, sex, smoking status, COPD status, asthma, tumor size, Charlson comorbidity index, nodal status, white cell count, creatinine, and hemoglobin.

Only variables that were statistically significant on multivariable analysis are presented.

The RRs for significant variables are in bold text.

**Appendix Table 3:** Interaction of Inhaled Corticosteroids and BEC on the risk of 90-day healthcare utilization in the study cohort

|  | **Risk Ratio (95% CI)** | **p-value** |
| --- | --- | --- |
| **BEC ≥ 200** | **2.51 (1.44-4.38)** | **0.006** |
| **ICS** | **1.72 (1.16-2.54)** | **0.001** |
| **BEC ≥ 200 * ICS** | **0.23 (0.05-1.01)** | **0.05** |

*Definition of abbreviations*: BEC = Blood eosinophil count (cells/µL); CI = Confidence Interval; ICS = Inhaled corticosteroids.

Appendix Table 4: Validation cohort characteristics and comparison between low and high blood eosinophils counts

| **Variable** | **Total N = 234** | **BEC < 200 n = 146** | **BEC ≥ 200 n = 88** | **p-value** |
| --- | --- | --- | --- | --- |
| Female sex | 111 (47.4%) | 68 (46.6%) | 43 (48.9%) | 0.734 |
| Age, median (IQR) | 71 (65-76) | 72 (66-76) | 70 (65-75) | 0.353 |
| Smoking |  |  |  |  |
| Never | 33 (14.1%) | 23 (15.8%) | 10 (11.4%) | 0.375 |
| Prior | 133 (56.8%) | 78 (53.4%) | 55 (62.5%) |  |
| Current | 68 (29.1%) | 45 (30.8%) | 23 (26.1%) |  |
| Charlson comorbidity index, median (IQR) | 4 (3-5) | 4 (3-5) | 3 (2-5) | 0.189 |
| COPD | 58 (24.7%) | 36 (24.7%) | 22 (25.0%) | 0.953 |
| Asthma | 9 (3.8%) | 3 (2.1%) | 6 (6.8%) | 0.066 |
| Inhalers |  |  |  |  |
| ICS use | 33 (14.1%) | 21 (14.4%) | 12 (13.6%) | 0.874 |
| LABA use | 40 (17.1%) | 25 (17.1%) | 15 (17.0%) | 0.988 |
| LAMA use | 32 (13.7%) | 22 (15.1%) | 10 (11.4%) | 0.424 |
| Tumor type |  |  |  |  |
| Squamous cell carcinoma | 51 (21.8%) | 31 (21.2%) | 20 (22.7%) | 0.789 |
| Adenocarcinoma | 183 (78.2%) | 115 (78.8%) | 68 (77.3%) |  |
| Tumor size |  |  |  |  |
| T1 | 154 (65.8%) | 93 (63.7%) | 61 (69.3%) | 0.532 |
| T2 | 79 (33.8%) | 53 (36.3%) | 27 (30.7%) |  |
| T3 | - | - | - |  |
| Nodal status |  |  |  |  |
| N0 | 211 (90.2%) | 135 (92.5%) | 76 (86.4%) | 0.129 |
| N1 | 23 (9.8%) | 11 (7.5%) | 12 (13.6%) |  |
| Procedure |  |  |  |  |
| Lobectomy | 208 (88.9%) | 131 (89.7%) | 77 (87.5%) | 0.683 |
| Segmentectomy | 12 (5.1%) | 7 (4.8%) | 5 (5.7%) |  |
| Wedge | 10 (4.3%) | 5 (3.4%) | 5 (5.7%) |  |
| Surgical approach |  |  |  |  |
| Open | 44 (18.8%) | 26 (17.8%) | 18 (20.5%) | 0.616 |
| VATS | 190 (81.2%) | 120 (82.2%) | 70 (79.5%) |  |

*Definition of abbreviations*: BEC = Blood eosinophil count (cells/µL); ICS = Inhaled corticosteroids; IQR = Interquartile range; LABA = Long-acting beta-agonist; LAMA = Long-acting muscarinic antagonist; SD = Standard Deviation; VATS = Video-assisted thoracoscopic surgery.

Appendix Table 5: Factors independently associated with 90-day healthcare utilization in the validation cohort

| Variable | | Unadjusted Risk Ratio  (95% CI) | p-value | Adjusted Risk Ratio  (95% CI) | p-value |
| --- | --- | --- | --- | --- | --- |
| **BEC, cells/µL*** | | **1.47 (1.07-1.80)** | **0.01** | **1.42 (1.10-1.94)** | **0.01** |
| **Smoking** | Current | 1 (Ref) | - | 1 (Ref) | - |
|  | Former | 0.54 (0.27-1.08) | 0.08 | **0.45 (0.21-0.98)** | **0.04** |
|  | Never | 0.48 (0.16-1.42) | 0.18 | 0.65 (0.19-2.20) | 0.49 |
| **CCI** | ≤ 1 | 1 (Ref) | - | 1 (Ref) | - |
|  | 2-3 | 2.31 (0.28-19.10) | 0.44 | 2.55 (0.30-22.00) | 0.39 |
|  | ≥ 4 | 6.25 (0.89-49.00) | 0.08 | **9.11 (1.09-76.40)** | **0.04** |

*Definition of abbreviations*: BEC = Blood eosinophil count (cells/µL); CCI = Charlson Comorbidity Index; CI = Confidence Interval.

*BEC was used as a continuous variable.

The RRs for significant variables are in bold text.

Adjusted for age, sex, smoking status, COPD status, asthma, CCI, tumor size, nodal status, white cell count, creatinine, and hemoglobin. Only those variables that were statistically significant on multivariable analysis are presented here.

**Appendix Table 6:** Adverse health outcomes risks by preoperative BEC threshold in the validation cohort

| **BEC, cells/µL** | **No. of patients (%)** | **Composite: Surgery LOS ≥7, ED/hospital readmission, or death by 90 days** | | **Surgery**  **LOS ≥ 7 days** | | **ED/hospital readmission**  **by postop day 90** | | **Death**  **by postop**  **day 90** | |
| --- | --- | --- | --- | --- | --- | --- | --- | --- | --- |
| < 200 | 146 (62%) | 64 (44%) | RR 1.12 | 51 (35%) | RR 1.17 | 23 (16%) | RR 2.04  P=0.031 | 1 (0.7%) | RR 8.68  P=0.050 |
| ≥ 200 | 88 (38%) | 41 (47%) |  | 34 (39%) |  | 24 (27%) |  | 5 (5.7%) |  |
| < 300 | 191 (82%) | 78 (41%) | RR 2.45  P=0.010 | 64 (34%) | RR 1.89 | 31 (16%) | RR 3.18  P=0.002 | 2 (1.0%) | RR 9.64  P=0.010 |
| ≥ 300 | 43 (18%) | 27 (63%) |  | 21 (49%) |  | 16 (37%) |  | 4 (9.3%) |  |

*Definition of abbreviations*: BEC = Blood eosinophil count (cells/µL); ED = Emergency department; LOS = Length of stay; RR=relative risk.

Postoperative adverse event risks and relative risks are summarized at various BEC thresholds.

**Appendix Figure 1:** Natural cubic spline function for eosinophil counts (10^9^ cells/L) as a continuous variable and the associated risk ratio of 90-day healthcare utilization.


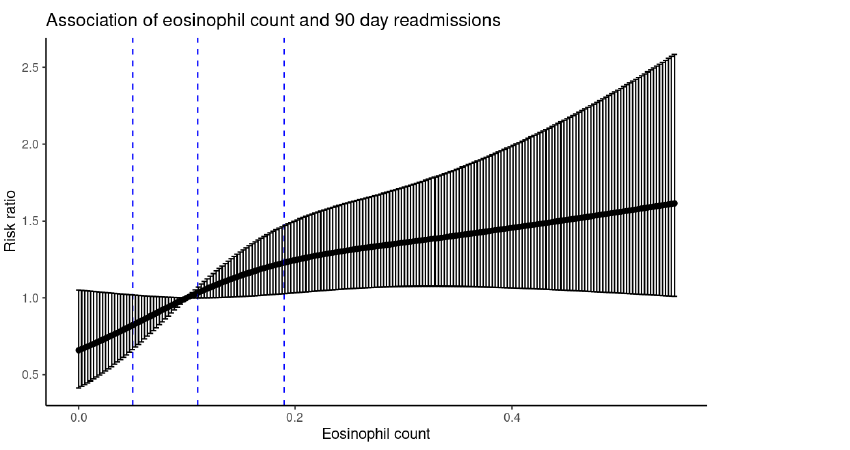


The knots are at 0.05, 0.11 and 0.19 cells x 10^9^/L (25th, 50th and 75th percentile).

**Appendix Figure 2:** Kaplan Meier curves of 1-year survival stratified by BEC ≥ and < 200 cells/µL in the main cohort


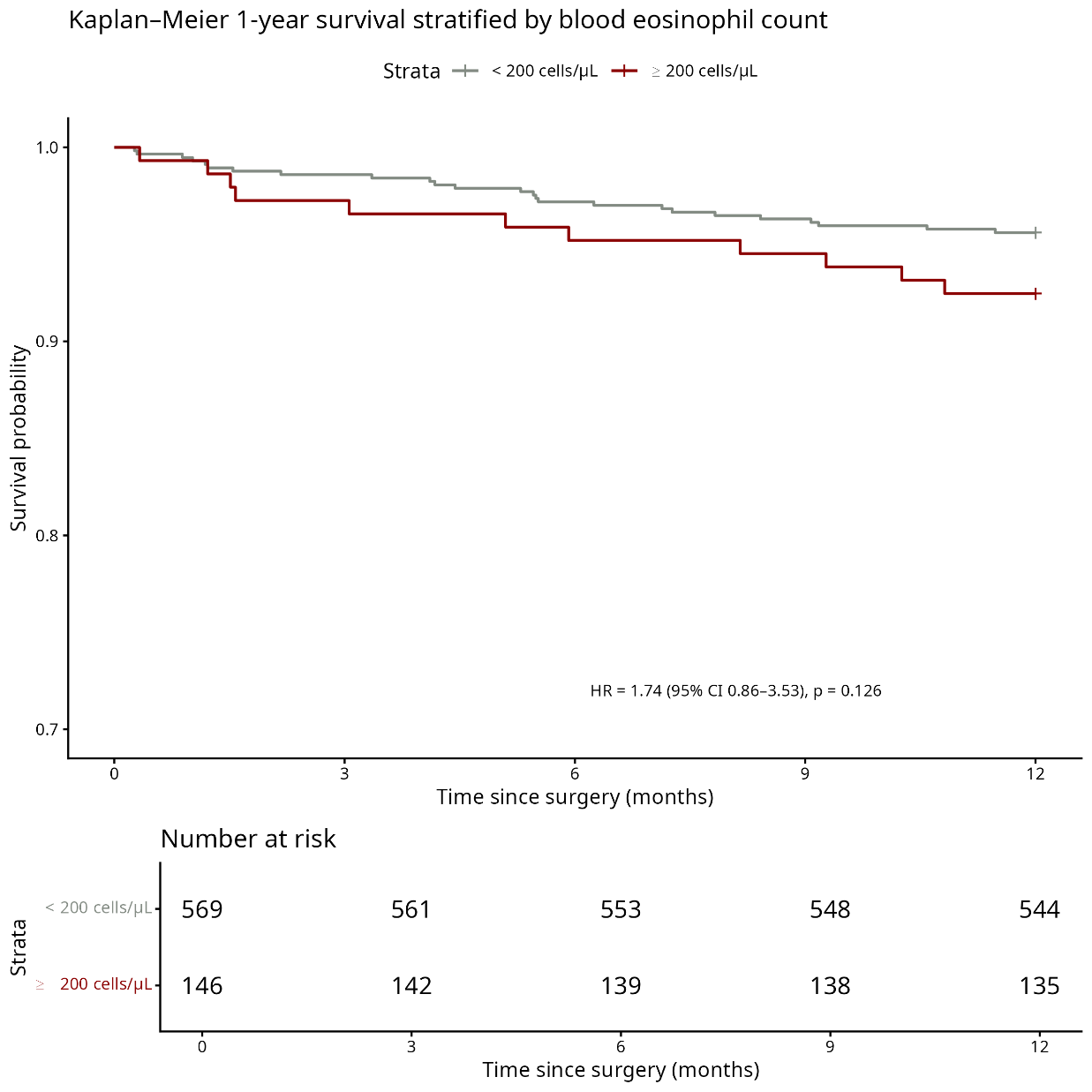

Supplement: Supplementary file 1 — Supplementary Material 1. [file 12931_2026_3672_MOESM1_ESM.docx]
